# Performance of the Kato-Katz method and real time polymerase chain reaction for the diagnosis of soil-transmitted helminthiasis in the framework of a randomised controlled trial: treatment efficacy and day-to-day variation

**DOI:** 10.1186/s13071-020-04401-x

**Published:** 2020-10-15

**Authors:** Ladina Keller, Chandni Patel, Sophie Welsche, Tobias Schindler, Eveline Hürlimann, Jennifer Keiser

**Affiliations:** 1 Swiss Tropical and Public Health Institute, Socinstrasse 57, 4051 Basel, Switzerland; 2 University of Basel, Petersplatz 1, 4051 Basel, Switzerland

**Keywords:** *Trichuris trichiura*, qPCR, Kato-Katz, Drug efficacy, Molecular diagnosis, Ivermectin, Soil-transmitted helminths, Diagnostic performance, Albendazole

## Abstract

**Background:**

Accurate, scalable and sensitive diagnostic tools are crucial in determining prevalence of soil-transmitted helminths (STH), assessing infection intensities and monitoring treatment efficacy. However, assessments on treatment efficacy comparing traditional microscopic to newly emerging molecular approaches such as quantitative Polymerase Chain Reaction (qPCR) are scarce and hampered partly by lack of an established diagnostic gold standard.

**Methods:**

We compared the performance of the copromicroscopic Kato-Katz method to qPCR in the framework of a randomized controlled trial on Pemba Island, Tanzania, evaluating treatment efficacy based on cure rates of albendazole monotherapy *versus* ivermectin-albendazole against *Trichuris trichiura* and concomitant STH infections. Day-to-day variability of both diagnostic methods was assessed to elucidate reproducibility of test results by analysing two stool samples before and two stool samples after treatment of 160 *T. trichiura* Kato-Katz positive participants, partially co-infected with *Ascaris lumbricoides* and hookworm, per treatment arm (*n* = 320). As negative controls, two faecal samples of 180 Kato-Katz helminth negative participants were analysed.

**Results:**

Fair to moderate correlation between microscopic egg count and DNA copy number for the different STH species was observed at baseline and follow-up. Results indicated higher sensitivity of qPCR for all three STH species across all time points; however, we found lower test result reproducibility compared to Kato-Katz. When assessed with two samples from consecutive days by qPCR, cure rates were significantly lower for *T. trichiura* (23.2 *vs* 46.8%), *A. lumbricoides* (75.3 *vs* 100%) and hookworm (52.4 *vs* 78.3%) in the ivermectin-albendazole treatment arm, when compared to Kato-Katz.

**Conclusions:**

qPCR diagnosis showed lower reproducibility of test results compared to Kato-Katz, hence multiple samples per participant should be analysed to achieve a reliable diagnosis of STH infection. Our study confirms that cure rates are overestimated using Kato-Katz alone. Our findings emphasize that standardized and accurate molecular diagnostic tools are urgently needed for future monitoring within STH control and/or elimination programmes.
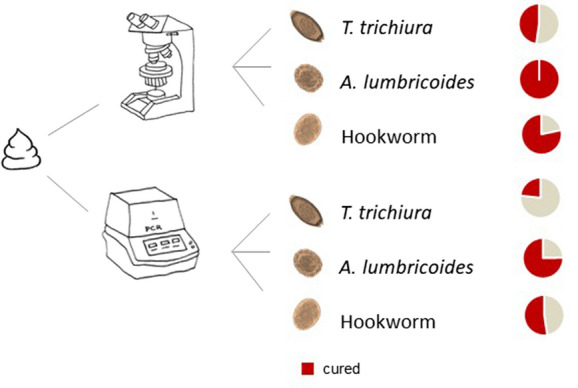

## Background

With an estimated 1.5 billion infections, the soil-transmitted helminths (STHs), namely *Ascaris lumbricoides*, *Trichuris trichiura* and the hookworms *Necator americanus* and *Ancylostoma duodenale*, are of enormous public health importance in subtropical and tropical regions, particularly amongst the most marginalized populations [1]. Diseases accompanying these infections can cause considerable burden manifested as malnutrition [2, 3], impairment in physical and cognitive development in children [4], reduction in work performance in adulthood [5] and adverse pregnancy outcomes [3, 6]. Preventive chemotherapy, the periodic large-scale administration of anthelminthic medicines to at-risk populations without prior diagnosis is the cornerstone of helminth control recommended by the World Health Organization (WHO). It is considered simple and cost-effective in its implementation and to have a strong impact on morbidity by decreasing the worm burden [7]. Accurate, scalable and sensitive diagnostic tools are crucial to assess and monitor treatment efficacy, prevalence and intensity of infection to guide future interventions, including the early detection of possible resistance development [8–13]. Cost-effective, sensitive techniques are paramount especially in areas of low endemicity, where a robust surveillance system is needed to approach and monitor elimination [13].

The microscopic Kato-Katz technique is a relatively simple and low-cost method recommended by the WHO for the detection of STH and other helminth eggs in faecal samples [14–16]. Consequently, it is widely used in randomised controlled trials (RCTs), epidemiological surveys and surveillance studies to determine the impact of STH interventions. Yet, the technique has considerable shortcomings. There is substantial variation in the readings, resulting from uneven distribution of eggs within a single stool sample (within sample variation), day-to-day fluctuations of egg excretion (between sample variations) and ultimately results depend on the readers’ skills and experience [17–20]. Most importantly, the Kato-Katz method may particularly miss low-intensity infections leading to underestimation of the actual prevalence, but in the case of efficacy trials artificially inflate cure rates (CRs) from undetected residual low-egg count infections post-treatment [21]. Moreover, expertise in microscopy is increasingly rare [22, 23].

Over the past few decades, molecular diagnostic methods have been developed for the use in human parasitology in order to increase sensitivity and specificity of the diagnosis of intestinal helminths. qPCR-based assays for the detection of helminth DNA or ribosomal RNA on faecal samples are the most widely used molecular methods [11, 23–25]. In recent years, further improvements of the DNA isolation step were made, and multiplex approaches have been developed to detect different parasite targets in a single procedure [17, 26]. Higher specificity and sensitivity of molecular diagnostics are generally observed in studies comparing the Kato-Katz thick smear stool examination to molecular methods (primarily qPCR), with rare exceptions [11, 20, 27–29]. The semi-quantitative output of PCR also reflects the amount of parasite DNA present, which could be of further interest as parasite burden rather than absence or presence of a STH infection is a key determinant of morbidity [17, 30]. Moreover, nucleic acid amplification may improve the detection in infections with low parasitic burden and has the ability to differentiate between morphologically identical species [31].

Evaluations on drug efficacy using molecular approaches are scarce, even though monitoring drug efficacy is of utmost importance for making treatment recommendations for novel therapies and in the light of possible upcoming anthelminthic resistance [32, 33]. Given the higher sensitivity and specificity of qPCR, the few available studies showed that treatment efficacy based on CRs is lower using qPRC detection compared to the microscopic Kato-Katz method. It is worth highlighting that STHs do not release eggs at a constant rate [34–36] and therefore, we hypothesize multiple collection of faecal samples might increase the sensitivity of qPCR.

The aim of the present study was to compare the performance of the microscopic Kato-Katz method and the molecular qPCR method for the diagnosis of soil-transmitted helminthiasis and its impact on treatment efficacy and day-to-day variation analysing two stool samples before and after treatment respectively. Stool samples were collected within the framework of a phase III, parallel group, double blind RCT assessing the safety and efficacy of the current standard treatment (albendazole) *versus* combination therapy (ivermectin-albendazole).

## Methods

### Trial design

Trial details are summarized in the published trial protocol [37] and in the trial registration (clinicaltrials.gov, reference: NCT03527732, date assigned: 17 May 2018). Participants were invited for clinical examination and treatment if found positive for *T. trichiura* infection in at least two slides of quadruple Kato-Katz thick smears with an infection intensity of at least 100 eggs per gram (EPG) of stool. The samples analysed in this work were collected at baseline and 14–21 days post-treatment between September 2018 and December 2018 in one of the three study settings, on Pemba Island, United Republic of Tanzania.

### Laboratory procedures

Two fresh morning stool samples were obtained from each participant within a maximum of 5 days using a door-to-door approach. Collected stool samples were kept in a cool box containing ice packs while being transported to the laboratory. Samples were examined with quadruplicate Kato-Katz microscopy within 24 h after collection for the detection of STH ova by experienced laboratory technicians following the WHO standard procedures [15]. An independent quality control of the Kato-Katz readings for *T. trichiura* and *A. lumbricoides* was conducted for 10% of the slides.

Stool samples of participants fulfilling eligibility criteria (minimal egg count for *T.* *trichiura* ≥ 100 EPG, 2 or more out of 4 Kato-Katz slides positive) and all identified STH egg negative participants (negative controls without any co-infection) were further processed. In total, 160 randomly selected *T. trichiura* Kato-Katz positive participants with complete aliquot pairs per treatment arm (*n* = 320) and 180 identified Kato-Katz helminth negative participants with two baseline aliquots were analysed. An aliquot of stool (~ 1 g) was mixed with 80% ethanol and preserved at 4 °C and shipped at room temperature to the Swiss Tropical and Public Health Institute (Swiss TPH) in Basel, Switzerland for subsequent qPCR analyses.

DNA extraction was performed using the QIAamp DNA Mini kit (Qiagen; Hilden, Germany) with slight modifications from the standard protocol validated and described by Kaisar et al. [17]. A multiplex real-time qPCR was used for simultaneous detection of *A. lumbricoides*, *T.* *trichiura*, *N. americanus*, *A.* *duodenale* and *Strongyloides stercoralis.* However, the latter parasite was not expected in these samples [38] but was placed together with the hookworm species in the same color channel, in case further specification would be of interest in a second round. Amplification consisted of 2 min at 50 °C, 10 min at 95 °C followed by 45 cycles of 15 s at 95 °C and 1 min at 58 °C. Testing was performed using CFX Maestro™ (Bio-Rad Laboratories, Inc, Hercules, CA, USA). qPCR plate planes were generated with a random distribution and balance; however, all samples of one participant were distributed within one plate to reduce between-plate variability and twelve worm-negative controls were placed in between. Four negative controls containing double-distilled water were randomly placed on each plate to ensure detection of confounding factors. For subsequent standardization of each plate, nine positive controls with rising plasmid concentrations (10^1^, 10^3^ and 10^5^ plasmids/µl) containing an insert with the sequence of the STH qPCR product were included in each amplification run. Standard curves were generated by plotting cycle threshold (Ct) values against the logarithm of starting DNA quantities.

The DNA amplification results of a serial 10-fold dilution series of the plasmids from each specimen were compared in separate reactions. Each dilution series was tested both with and without the other target DNAs to assess the assay’s ability to detect mixed infections. The details of all primers and detection probes (Eurofin Genomics, Ebersberg, Germany) and the concentrations of the qPCR using TaqMan GeneExpression MasterMix (ThermoFischer, Switzerland) are presented in the supplementary data (Additional file 1: Tables S1, Additional file 2: Table S2). Extraction of DNA, preparation of the master mix and handling of qPCR products were all performed in different rooms to prevent contamination.

### Data preparation

All qPCR assays with an observed copy number above zero were considered positive. All qPCR assays for which no amplification curves were obtained, were considered negative (equalling zero copy numbers). Kato-Katz results were calculated as mean egg counts of the two slides of each time point assessment (baseline day 1, baseline day 2, follow-up day 1 and follow-up day 2) and samples considered positive if at least 0.5 eggs per sample were identified. Data of the amplification curves were cleaned and standardised according to the standard curves with CFX Maestro™ Software and then uploaded to ELIMU-MDx, an open-source platform for storage, management and analysis of diagnostic qPCR data [39]. Subsequent statistical analyses were conducted using Stata IC15 (StataCorp., College Station, TX).

The Ct value is defined as the number of qPCR cycles needed for the detection of fluorescence signal of the amplified products to pass the fixed threshold value. Accordingly, exceeding that threshold can be interpreted as the earliest qPCR cycle at which point a sample’s amplification product is statistically different from the background fluorescence [40]. Consequently, higher quantities of helminth DNA are inversely proportional and thus, result in lower Ct values and *vice versa* [23]. Amplification curves not following a sigmoidal shape were considered as negative results interpreting these signals as unspecific background noise. Samples below Ct value of 15 were excluded for that species, as the range of standards tested and detected was above Ct 15. Data of the amplification curves were then translated into copies/µl DNA by inserting the average slopes and y-intercepts for each quantified target of the standard curves into a linear equation. Thus, the cycle cut-off points vary for each quencher, depending on the calibration curves obtained. This procedure was done to avoid choosing an arbitrary Ct cutoff which is known to not be ideal, by either being too low (eliminating valid results) or being too high (increasing false-positive results) [40].

### Statistical analysis

Based on available summarised efficacy measures from a recent review [41] and the published literature, the CR of albendazole against *T.* *trichiura* was assumed to be 30% compared to 50% in the ivermectin-albendazole treatment regimen according to Kato-Katz. Moreover, the correlation between the two diagnostic test results was assumed to be 0.6. A sample size of 320 Kato-Katz *T.* *trichiura* positives (160/treatment arm) was chosen to detect a 10% difference in CRs against *T. trichiura* between Kato Katz and qPCR with a power of 80% assuming a two-sided type 1 error of 5%. An additional subsample of 320 Kato-Katz negatives (1:1 ratio to the positives) was aimed for to determine the sensitivity of qPCR *versus* Kato-Katz. Since STH infections are staggeringly prevalent on Pemba Island, we only found 180 helminth negative individuals within the screening phase.

#### Correlation between microscopic egg count and DNA copy number

Correlation between copy numbers/µl DNA according to qPCR and egg count numbers derived by the Kato-Katz thick smear method were assessed as a base for sensitivity and specificity estimates. Spearman’s rank correlation coefficients *r*_*S*_ were calculated for each species and each time point among the samples, which were positive according to both diagnostic methods to assess potential correlation. The degree of agreement was categorised as “poor” (*r*_*S*_ < 0.2), “fair” (0.2 ≤ *r*_*S*_ < 0.4), moderate (0.4 ≤ *r*_*S*_ < 0.6) good (0.6 ≤ *r*_*S*_ < 0.8) and very good (*r*_*S*_ ≥ 0.8) agreement [42].

#### Diagnostic method variability between samples

To assess the agreement of test results between baseline day 1 and day 2 and follow-up day 1 and day 2, for qPCR and Kato-Katz, Spearman’s rank correlation of copy numbers and egg counts, respectively, was performed among all samples, which were found positive according to both techniques. An alternative assessment was based on Cohen’s Kappa, comparing positivity of qPCR and Kato-Katz between baseline day 1 and day 2 and between follow-up day 1 and 2, including negative and positive test results. The *κ*-statistics categorised in the same way as the rank correlation coefficients *r*_*S*_.

#### Overall sensitivity of Kato-Katz and qPCR

The sensitivity was determined assuming a 100% sensitivity and specificity of each diagnostic method, as disclosed by the morphology of the eggs or by the species-specific qPCR assays. Sensitivities of qPCR relative to Kato-Katz and *vice versa* were calculated for baseline and follow-up separately and for both time points combined. A qPCR test at baseline or follow-up was considered positive if at least one of the two samples taken on the respective consecutive days provided a positive result. The 95% confidence intervals for sensitivities across both time points were computed using a logistic regression model with robust standard errors adjusting for longitudinal correlations of test results within individuals.

#### Cure rates according to Kato-Katz and qPCR

CRs were calculated as the proportion of participants negative for infection (EPG or transformed DNA copy equalling zero) in both follow-up stool samples among those who were positive at baseline in any sample. Moreover, CRs assessed by qPCR were also calculated considering only the first follow-up sample to assess if test result reliability affects CRs. Logistic regression models were used to compare CRs between different treatment arms. Comparisons of CRs between the qPCR and the Kato-Katz method also required the use of robust standard errors adjusting for correlations of outcomes within subjects. Statistical significance of observed differences or associations was defined as a two-sided *P*-value smaller than 0.05.

## Results

**Fig. 1:**
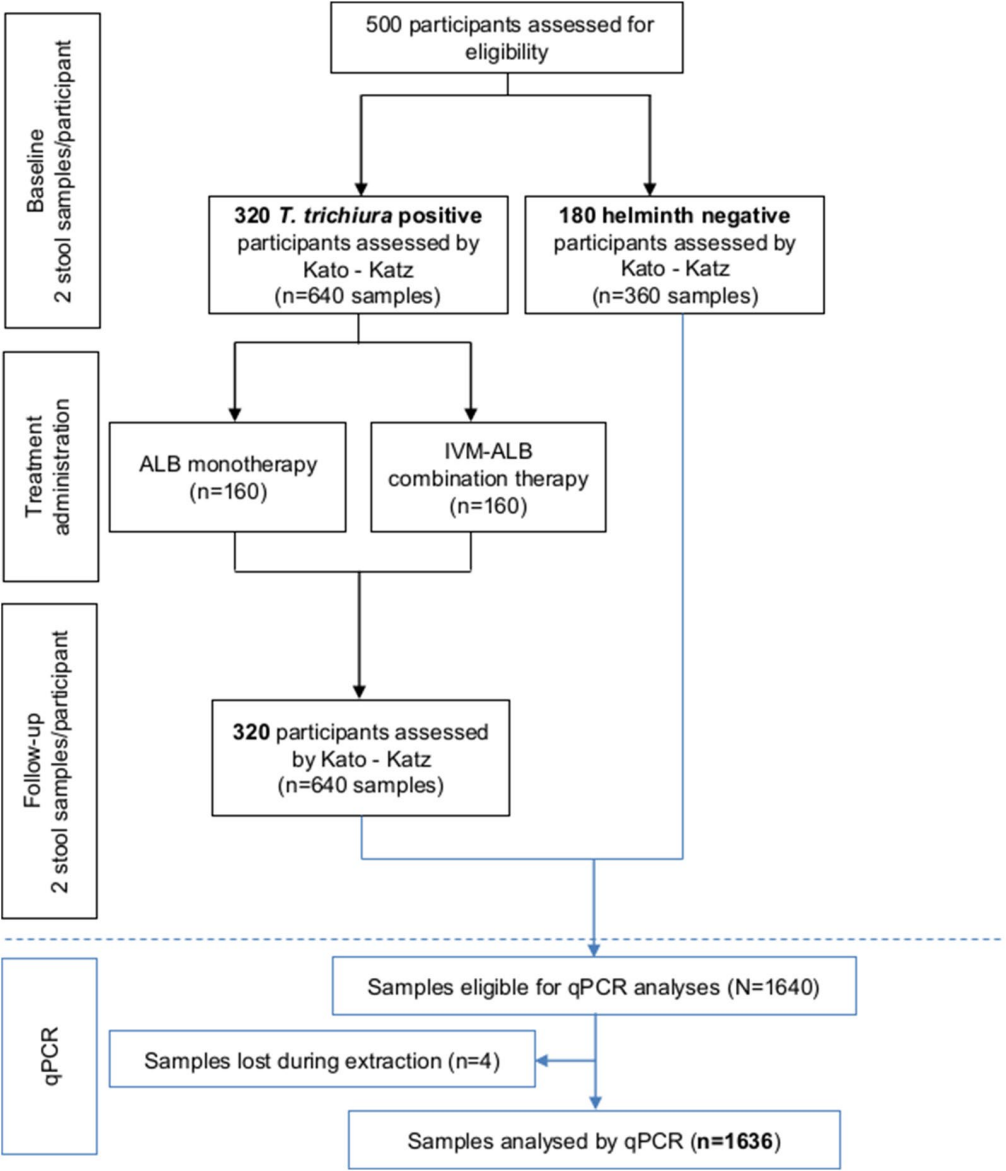
Study design. *Abbreviations*: *T. trichiura, Trichuris trichiura;* ALB, albendazole; IVM-ALB, ivermectin-albendazole; qPCR, quantitative polymerase chain reaction

Two stool samples of 320 *T. trichiura* positive participants, partially co-infected with *A. lumbricoides* and hookworm at baseline and two stool samples 14–21 days post-treatment were processed by both, Kato-Katz and qPCR method. As negative controls, two faecal samples of 180 individuals negative for STH eggs as assessed by Kato-Katz were analysed (Fig. 1).

### Overall positivity agreement according to Kato-Katz and qPCR for all four examination time points pooled

In total, 1020 samples were positive for *T. trichiura* according to Kato-Katz and 1134 were positive for *T. trichiura* according to qPCR. There were 394 samples with discordant results, 254 where only the qPCR-result was positive and 140 where only the Kato-Katz result was positive. Results for *A. lumbricoides* and hookworm showed even more pronounced differences, with most discordant tests being positive for qPCR and negative for Kato-Katz (Table 1).

**Table 1:** Positivity agreement according to Kato-Katz and qPCR for all four-examination time points pooled (*n* = 1636) for *T. trichiura*, *A. lumbricoides* and hookworm

		No. of qPCR negatives (%)	No. of qPCR positives (%)	Total (%)
*T. trichiura*	Kato-Katz negative	362 (58.8)	254 (41.2)	616 (100)
	Kato-Katz positive	140 (13.7)	880 (86.3)	1020 (100)
	Total	502 (30.7)	1134 (69.3)	
*A. lumbricoides*	Kato-Katz negative	1236 (84.1)	233 (15.9)	1469 (100)
	Kato-Katz positive	28 (16.8)	139 (83.2)	167 (100)
	Total	1264 (77.3)	372 (22.7)	
Hookworm	Kato-Katz negative	1369 (89.5)	160 (10.5)	1529 (100)
	Kato-Katz positive	18 (16.8)	89 (83.2)	107 (100)
	Total	1387 (84.8)	249 (15.2)	

*Abbreviations*: *T. trichiura*, *Trichuris trichiura*; *A. lumbricoides*, *Ascaris lumbricoides*

### Correlation between microscopic egg count and DNA copy number

For *T. trichiura,* correlation of positive parasite loads assessed by the two diagnostic methods was moderate (*r*_*S*_ = 0.47, 0.45, 0.47, 0.51) for all four time points (baseline day 1, baseline day 2, follow-up day 1, follow-up day 2). For *A. lumbricoides,* correlation was moderate (*r*_*S*_ = 0.55) for the first and fair (*r*_*S*_ = 0.36) for the second baseline examination time point. It was not possible to apply the Spearman’s rank correlation test for the follow-up examination time points, due to only a few Kato-Katz positive samples. For hookworm, correlation was moderate (*r*_*S*_ = 0.48, 0.47) for both baseline examination time points and the first follow-up examination time point (*r*_*S*_ = 0.49), whereas the second follow-up time point showed fair agreement (*r*_*S*_ = 0.26) (Table 2). Both correlations had *P*-values > 0.2 and them being chance results can therefore not be ruled out.

**Table 2:** Spearman’s rank correlations between EPG and DNA copy numbers for each time point among positive tests for each STH species

		No. of positive test results^a^	*ρ*	*P*-value
*T. trichiura*	Baseline day 1	272	0.47	< 0.001
	Baseline day 2	267	0.45	< 0.001
	Follow-up day 1	175	0.47	< 0.001
	Follow-up day 2	166	0.51	< 0.001
*A. lumbricoides*	Baseline day 1	68	0.55	< 0.001
	Baseline day 2	69	0.36	< 0.001
	Follow-up day 1	2	^b^–	^b^–
	Follow-up day 2	0	^b^–	^b^–
Hookworm	Baseline day 1	35	0.48	< 0.001
	Baseline day 2	34	0.47	< 0.001
	Follow-up day 1	7	0.49	0. 2682
	Follow-up day 2	13	0.26	0.3833

^a^Positive according to both diagnostic methods

^b^–, Sample size is not sufficient for Spearman’s rank correlation analysis

*Abbreviations*: *ρ*, Spearman’s rank correlation coefficient; *T. trichiura*, *Trichuris trichiura*; *A. lumbricoides*, *Ascaris lumbricoides*

### Diagnostic method variability between samples

To assess the variability within one diagnostic method, the agreements between baseline day 1 and day 2 as well as between follow-up day 1 and day 2 were calculated using Spearman’s rank correlation, including all positive test results according to both techniques. qPCR showed moderate agreement for *T. trichiura* (*r*_*S*_ = 0.51) and good agreement for *A. lumbricoides* (*r*_*S*_ = 0.62) and hookworm (*r*_*S*_ = 0.64) at baseline. At follow-up, moderate agreement for *T. trichiura* (*r*_*S*_ = 0. 45) and hookworm (*r*_*S*_ = 0.49) and good agreement for *A. lumbricoides* (*r*_*S*_ = 0.71) was found. Kato-Katz results showed moderate agreement for *T. trichiura* (*r*_*S*_ = 0.49) and good agreement for *A. lumbricoides* (*r*_*S*_ = 0.65) and hookworm (*r*_*S*_ = 0.70) between the two baseline samples. Moderate agreement between the follow-up samples was shown for *T. trichiura* (*r*_*S*_ = 0.60) and poor agreement for hookworm (*r*_*S*_ = − 0.55) was observed. Agreement could not be assessed for *A. lumbricoides* at follow-up as no samples were positive by both methods (Table 3).

**Table 3:** Spearman’s rank correlations of copy numbers (qPCR) resp. egg counts (Kato-Katz) between baseline day 1 and day 2 and between follow-up day 1 and day 2 for qPCR and Kato-Katz (restricted to positive test results)

	Baseline (day 1 *vs* day 2)			Follow-up (day 1 *vs* day 2)		
	No. of positive test results	*ρ*	*P*-value	No. of positive test results	*ρ*	*P*-value
qPCR						
*T. trichiura*	293	0.51	< 0.001	190	0.45	< 0.001
*A. lumbricoides*	114	0.62	< 0.001	6	0.71	0.1108
Hookworm	57	0.64	< 0.001	22	0.49	0.0214
Kato-Katz						
*T. trichiura*	302	0.49	< 0.001	166	0.6	< 0.001
*A. lumbricoides*	77	0.65	< 0.001	0	–	–
Hookworm	32	0.7	< 0.001	6	− 0.55	0.2574

*Abbreviations*: *ρ*, Spearmanʼs rank correlation coefficient; *T. trichiura*, *Trichuris trichiura*; *A. lumbricoides*, *Ascaris lumbricoides*

Additionally, Cohen’s Kappa was used to assess reproducibility including positive and negative test results between baseline day 1 and day 2 and between follow-up day 1 and day 2. qPCR test results between baseline and between follow-up samples showed moderate agreement for *T. trichiura* (κ = 0.57, 0.42) and hookworm (κ = 0.58, 0.52). For *A. lumbricoides*, qPCR showed good agreement between baseline (κ = 0.63), but only poor agreement (κ = 0.1) between follow-up samples. Kato-Katz showed very good agreement for *T. trichiura* (κ = 0.92) and *A. lumbricoides* (κ = 0.93) and good agreement for hookworm (κ = 0.72) between baseline samples. The agreement between follow-up samples was moderate for *T. trichiura* (κ = 0.56) and hookworm (κ = 0.56). However, there was poor agreement (κ = − 0.004) for *A. lumbricoides,* possibly because only a few positive samples were found by the Kato-Katz technique (Table 4).

**Table 4:** Cohens Kappa for positivity of qPCR resp. Kato-Katz between baseline day 1 and day 2 and between follow-up day 1 and day 2

	Baseline (day 1 *vs* day 2)				Follow-up (day 1 *vs* day 2)			
	Observed agreement (%)	Expected agreement^a^ (%)	*κ*	*P*-value	Observed agreement (%)	Expected agreement (%)	*κ*	*P*-value
qPCR								
*T. trichiura*	81.4	56.4	0.57	< 0.001	76.4	59.3	0.42	< 0.001
*A. lumbricoides*	84.4	57.5	0.63	< 0.001	83.5	81.8	0.1	0.043
Hookworm	88.2	71.8	0. 58	< 0.001	89.9	78.9	0.52	< 0.001
Kato-Katz								
*T. trichiura*	96.4	53	0.92	< 0.001	79.4	53.2	0.57	< 0.001
*A. lumbricoides*	98	72.6	0.93	< 0.001	99.1	99.1	0	0.5319
Hookworm	95.6	84.3	0.72	< 0.001	97.2	93.6	0.56	< 0.001

^a^Percentage of agreement expected based on chance alone (i.e. if the two results compared were completely uncorrelated)

*Abbreviations*: κ, Cohen’s kappa coefficient; *T. trichiura*, *Trichuris trichiura*; *A. lumbricoides*, *Ascaris lumbricoides*

Overall, qPCR results showed greater variability between both baseline and follow-up samples compared to Kato-Katz, especially when looking only at the positivity and negativity of samples and using Cohen’s kappa coefficient.

### Overall sensitivity of Kato-Katz and qPCR

Across all comparisons between the two methods, the sensitivity of qPCR in detecting positive samples according to Kato-Katz was higher than the respective sensitivity of Kato-Katz in detecting positive samples according to qPCR (Table 5). When pooling baseline and follow-up results, the pooled sensitivity of qPCR relative to Kato-Katz was 93.7% for *T. trichiura*, 84.4% for *A. lumbricoides* and 88.4% for hookworm, while the sensitivity of Kato-Katz relative to qPCR was 79.5%, 30.4% and 35.9% for *T. trichiura*, *A. lumbricoides* and hookworm, respectively. Interestingly, the sensitivity of Kato-Katz relative to qPCR significantly dropped from 38.5% (31.6–45.8) at baseline to 3.4% (0.4–11.9) at follow-up in the case of *A. lumbricoides*.

**Table 5:** Mutual sensitivities calculated for combined Kato-Katz and combined qPCR for *T. trichiura*, *A. lumbricoides* and hookworm at baseline and 14–21 days follow-up

		Sensitivity (%) (95% CI)		Sensitivity (%) (95% CI)	*P*-value*		Pooled analysis (%) (95% CI)
	*n*	Baseline	*n*	Follow-up		*n*	Baseline + Follow-up
*T. trichiura*							
Kato-Katz *vs* PCR (= Ref)	386	78.2 (73.8–82.2)	264	81.4 (76.2–85.9)	0.35	650	79.7 (76.4–82.6)
PCR *vs* Kato-Katz (= Ref)	320	94.4 (91.3–96.6)	232	92.7 (88.5–95.7)	0.42	552	93.7 (91.3–95.4)
*A. lumbricoides*							
Kato-Katz *vs* PCR (=Ref)	192	38.5 (31.6–45.8)	58	3.4 (0.4–11.9)	< 0.001	250	30.4 (25.0–36.4)
PCR *vs* Kato-Katz (=Ref)	87	85.1 (75.8–91.8)	3	66.7 (9.4–99.2)	0.41	90	84.4 (75.4–90.6)
Hookworm							
Kato-Katz *vs* PCR (=Ref)	116	39.7 (30.7–49.2)	54	27.8 (16.5–41.6)	0.13	170	35.9 (29.0–43.4)
PCR *vs* Kato-Katz (=Ref)	54	85.2 (72.9–93.4)	15	100 (78.2–100)	0.19	69	88.4 (78.5–94.1)

*P-values were calculated for the difference in the respective sensitivity measure between the baseline and follow-up examination time point using logistic regression models with robust standard errors adjusting for longitudinal correlations of test results within individuals

*Note*: The number of observations (*n*) is determined by samples found positive by the method considered as reference. The reference method was assumed to be the gold standard, i.e. with a 100% sensitivity and specificity, when assessing sensitivities of the other method

*Abbreviations*: Ref, reference; *T. trichiura*, *Trichuris trichiura*; *A. lumbricoides*, *Ascaris lumbricoides*

### Cure rates according to Kato-Katz and qPCR

Cure rates for *T. trichiura* were significantly lower with albendazole monotherapy than with combination therapy (ivermectin-albendazole) with both diagnostic methods, while CRs were comparable for *A. lumbricoides* and hookworm. CRs of the combination therapy according to Kato-Katz were slightly higher (46.8 *vs* 36.4%, 100 *vs* 85.7%, 78.3 *vs* 71.4%) for *T. trichiura*, *A. lumbricoides* and hookworm, respectively, when only the first qPCR stool sample was considered. However, CRs according to Kato-Katz were significantly higher than CRs based on qPCR (46.8 *vs* 23.2%, 100 *vs* 75.3%, 78.3 *vs* 52.4%) for *T. trichiura*, *A. lumbricoides* and hookworm, respectively, when considering an additional second qPCR stool sample for the combination therapy. This was also the case for *A. lumbricoides* (95.0 *vs* 77.5%) with albendazole, while the CRs were comparable for *T. trichiura* (6.3 *vs* 5.3%) and hookworm (73.3 *vs* 66.7%).

Odds ratios (ORs) were calculated for the combination therapy (ivermectin-albendazole) compared to albendazole. The odds of being cured was significantly higher under combination therapy as compared to monotherapy in *T.* *trichiura* positives irrespective of the diagnostic approach and the amount of qPCR follow-up stool samples. CRs and ORs according to Kato-Katz and qPCR are listed in Table 6.

**Table 6:** Comparison of efficacy in terms of Cure Rates (CRs) and Odds Ratio (OR) for being cured between treatment arms (albendazole *vs* ivermectin-albendazole), by diagnostic approach (Kato-Katz on samples of day 1 and 2 *vs* qPCR on first day sample only and qPCR on samples of day 1 and 2)

	Kato-Katz			qPCR 1st Follow-up sample			qPCR 1st and 2nd Follow-up samples		
	ALB	IVM-ALB	*P*-value^a^	ALB	IVM-ALB	*P*-value^a^	ALB	IVM-ALB	*P*-value^a^
*T. trichiura*									
No. cured/No. positive BL	10/160	75/160		8/150	55/151		8/150	35/151	
Cure rates, % (95% CI)	6.3 (3.0–11.2)	**46.8 (39.7–55.9)**		13.3 (8.3–19.8)	36.4 (28.8–44.6)		5.3 (2.3–10.2)	**23.2 (16.7–30.7)**	
OR_cure_ (95% CI)	1	13.7 (6.7–28.0)	< 0.001	1	3.7 (2.1–6.6)	< 0.001	1	5.4 (2.4–12.0)	< 0.001
*A. lumbricoides*									
No. cured/No. positive BL	38/40	47/47		64/71	66/77		55/71	58/77	
Cure rates, % (95% CI)	**95 (83.1–99.4)**	**100 (92.5–100)**		90.1 (80.7–95.9)	85.7 (75.9–92.6)		**77.5 (66.0–86.5)**	**75.3 (64.2–84.4)**	
OR_cure_ (95% CI)	1	NA (0.62, –>)	0.21	1	0.6 (0.2–1.8)	0.41	1	0.6 (0.2–1.3)	0.76
Hookworm									
No. cured/No. positive BL	22/30	18/23		32/42	30/42		28/42	22/42	
Cure rates, % (95% CI)	73.3 (54.1–87.7)	**78.3 (56.3–92.5)**		76.2 (60.5–87.9)	71.4 (55.4–84.3)		66.7 (50.5–80.4)	**52.4 (36.4–68.0)**	
OR_cure_ (95% CI)	1	1.3 (0.4–4.7)	0.68	1	0.8 (0.3–2.0)	0.62	1	0.9 (0.4–1.9)	0.184

^a^*P*-values of the odds ratio (OR) for being cured between albendazole monotherapy (ALB) and ivermectin-albendazole (IVM-ALB) derived from logistic regression models

*Note*: CRs in bold highlight significant differences (*P* < 0.05) between CRs assessed by Kato-Katz and two qPCR samples in the respective treatment arm (i.e. ALB or IVM-ALB)

*Abbreviations*: BL, baseline; NA, not applicable; *T. trichiura*, *Trichuris trichiura*; *A. lumbricoides*, *Ascaris lumbricoides*

## Discussion

We evaluated the diagnostic performance of qPCR compared to standard Kato-Katz microscopy for the diagnosis of STHs and the resulting treatment efficacies by both methods. This study was done within the framework of a phase III, parallel group, double blind RCT assessing the efficacy and safety of the current standard treatment (albendazole) *versus* a combination therapy of ivermectin-albendazole. This was the first study analysing two stool samples before and two stool samples after treatment of each participant to assess diagnostic method variability between samples to elucidate reproducibility of test results of both diagnostic methods. Moreover, we present data on the efficacy of the most promising therapy to date for treating STH infections, ivermectin-albendazole, based on molecular diagnosis.

An interesting finding from the comparison of these two diagnostic methods is that an additional 41.2% of microscopy-negative samples were found *T. trichiura-*positive when assessed by qPCR. We hypothesise that lower DNA loads found in Kato-Katz-negative samples reflect higher detection rates by qPCR due to a higher sensitivity rather than lower specificity of the qPCR assays as remaining DNA of already dead worms or eggs can still act as a template DNA during qPCR [43].

We found that qPCR results indicate higher sensitivity for all species across all examination days compared to Kato-Katz, which substantiates previous findings [17, 27, 30, 34, 44–46]. Interestingly, follow-up Kato-Katz results differ significantly compared to the baseline results in the case of *A. lumbricoides*, indicating a time-dependent difference. The very low number of Kato-Katz positive test results post-treatment show the difficulty of detecting low *A. lumbricoides* worm burden by use of Kato-Katz, while qPCR was able to detect a considerable number of treatment failures.

Of note, the eligibility criteria (minimal *T. trichiura* egg count ≥ 100 EPG, 2 or more out of 4 Kato-Katz slides positive) for trial inclusion did not consider low *T. trichiura* infection intensities. Interestingly we observed that qPCR positivity of Kato-Katz-negative samples (EPG = 0) at baseline was lower compared to follow-up, implying higher sensitivity for qPCR when assessing low infection intensities. However, interpretation requires caution, as it is unclear how long residual DNA persists after parasite clearance leading to false-positive qPCR results [18, 47].

Our results highlight, that the combination therapy (ivermectin-albendazole) shows a significantly better efficacy compared to the monotherapy for *T. trichiura* with both diagnostic methods, while CRs were comparable for *A. lumbricoides* and hookworm. However, the observed low to moderate CRs for ivermectin-albendazole with qPCR (23.2% for *T. trichiura*, 75.3% for *A. lumbricoides* and 52.4% for hookworm) are far from benchmark target product profiles for anthelminthic drug candidates and combinations and highlight the need to develop novel efficacious treatments. A particularly striking difference in CRs of the combination chemotherapy (and for *A. lumbricoides* after albendazole treatment) between Kato-Katz and qPCR was observed when two qPCR samples were considered post-treatment. These results also stress to analyse two qPCR samples post-treatment in clinical trials to elucidate the true efficacy of treatments.

Although hypothesised, we only observed a fair to moderate agreement between microscopic egg count and DNA copy, which is in agreement with findings from Barda et al. [33]. Our results do not corroborate the observation of Mejia et al. [18], who found a significant good correlation (*r* = 0.7) between egg counts measured by the coprological Kato-Katz method and the DNA quantified by qPCR for *A. lumbricoides* and *T.* *trichiura*. The reason for this discrepancy is not entirely clear, but one partial explanation could be that only a few *A. lumbricoides-* or hookworm-positive samples were found post-treatment according to Kato-Katz in our study and that infection intensities were relatively low in these participants. As there is no strong correlation between egg counts and DNA copy number, finding a real gold standard for practical use remains a profound challenge, hampering the comparison of STH diagnostic tools.

As egg excretion is highly variable over time, there is considerable variation in EPG of faecal samples collected on consecutive days [34–36]. It is well known that Kato-Katz shows improved sensitivity when performed on several samples on different days [48, 49]. We observed that Kato-Katz showed very good agreement for *T. trichiura* and *A. lumbricoides* and good agreement for hookworm at baseline between day 1 and day 2, whereas qPCR only showed good agreement between day 1 and day 2 for *A. lumbricoides*, but not for *T. trichiura* and hookworm, indicating lower test result reproducibility of the qPCR method. This apparent high correlation of Kato-Katz test results might be explained by the laboratory technician’s skills, as the same well-trained microscopists were reading the stool samples every day, in addition to rather high *T. trichiura* parasite load (EPG ≥ 100 as inclusion criterion). The reason for the surprisingly low qPCR test reproducibility is not entirely clear; however, Pilotte et al. [50] found that common qPCR assays make use of sub-optimal target sequences limiting detection and species-specificity. Another explanation could be that, in contrast to bacteria and viruses, isolation of parasite DNA out of faecal samples is a challenging process as the wall of helminth eggs is difficult to lyse and thus several additional steps are needed to achieve release of nucleic acids [12, 51, 52]. Moreover, we based our analyses on DNA copies/µl as qPCR parameter for infection intensity, while consensus has not been reached on the optimal qPCR parameter with regard to reliability and reproducibility assessment.

We are aware that a number of limitations might have influenced the results obtained. The sample input volumes of the Kato-Katz assays are considerably larger than those of the qPCR assays, which might substantially increase sensitivity for Kato-Katz given the stochastic distribution of eggs in stool samples. On the other hand, increasing the stool volume for DNA extraction would not be possible, as faecal specimens contain various substances acting in a qPCR inhibitory manner [53]. Another limitation is that, in contrast to Kato-Katz, qPCR samples were not analysed in duplicates. Further research needs to be performed to elucidate if and for how long residual DNA may persist after parasite clearance as this might lead to false-positive qPCR results post-treatment [18, 29, 47]. Furthermore, we fully agree with Levecke et al. [54], that an agreement on an absolute universal unit for qPCR is needed to establish best comparison parameters for these two diagnostic methods. Lastly, it is important to note, that the standardisation and adherence to one approved protocol would help to achieve more readily comparable results between different research laboratories.

## Conclusions

The sensitive and scalable nature of qPCR makes its usage in large-scale diagnosis of intestinal helminths appealing over the rather operator-dependent microscopic method. DNA samples can be stored for further use, such as genetic characterisation and molecular typing, which might be of interest in surveillance studies to detect sporadic and focal infections or to monitor disease recrudescence. The evidence from this study implies statistically lower CRs (the primary outcome of this trial) for the combination therapy (ivermectin-albendazole) for all three species when assessed with two qPCR samples compared to Kato-Katz. Thus, it underlines the importance of the need for standardised and accurate molecular diagnostic tools, which are applicable in peripheral field settings, for future monitoring within STH control and/or elimination programmes and for developing novel efficacious treatments. This study has revealed for the first time, that qPCR test results show greater between day variability for baseline as well as post-treatment samples compared to Kato-Katz calling for a multi-sample analysis approach in order to improve qPCR-based diagnosis. This is of particular importance for studies aiming at assessing accurate disease prevalence as well as treatment efficacy. However, it needs to be carefully evaluated if the obtained higher sensitivity comes at the cost of the lower test reproducibility and how important this finding is in the context of preventive chemotherapy and the surveillance of low prevalence settings. Daily stool sample analyses to monitor dynamics of DNA copy numbers over a longer period post-treatment using the qPCR method might be one way forward to answer this question.

## Supplementary information

**Additional file 1: Table S1.** Primers and probes used to identify the different helminth species.

**Additional file 2: Table S2.** GeneExpression MasterMix.

## Data Availability

Data supporting the conclusions of this article are included within the article and its additional files. The datasets used and analysed during the present study are available from the corresponding author upon reasonable request.

## Abbreviations

CI: Confidence intervals
CR: Cure rates
C_t_: Cycle threshold
EKNZ: Ethikkommission Nordwest-und Zentralschweiz
EPG: Eggs per gram of stool
OR: Odds ratio
qPCR: Quantitative polymerase chain reaction
RCT: Randomized controlled trial
STH: Soil-transmitted helminths
Swiss TPH: Swiss Tropical and Public Health Institute
WHO: World Health Organization
ZAMREC: Zanzibar Medical Resarch and Ethics Committee
