# Supplementary material for: Performance of the Kato-Katz method and real time polymerase chain reaction for the diagnosis of soil-transmitted helminthiasis in the framework of a randomised controlled trial: treatment efficacy and day-to-day variation
Source: Parasit Vectors. 2020 Oct 15;13:517. doi: 10.1186/s13071-020-04401-x (PMC7558729; doi:10.1186/s13071-020-04401-x)
Supplement: Supplementary file 1 — Additional file 1: Table S1. Primers and probes used to identify the different helminth species. [file 13071_2020_4401_MOESM1_ESM.docx]

**Additional file 1: Table S1. Primers and probes used to identify the different helminth species.**

| **Soil Transmitted Helminths (STH)** |  |  |  |
| --- | --- | --- | --- |
| **Species , target gene, product size** | **Primer name** | **Dye and Quencher** | **Sequence 5ʼ-3ʼ** |
| ***Ascaris lumbricoides* qPCR** | Asca_F |  | GTAATAGCAGTCGGCGGTTTCTT |
| Target: ITS, Size: 88bp | Asca_R |  | GCCCAACATGCCACCTATTC |
|  | Asca_P | CY5-BHQ2 | TTGGCGGACAATTGCATGCGAT |
| ***Trichuris trichiura* qPCR** | Trich_F |  | TTGAAACGACTTGCTCATCAACTT |
| Target: 18S, Size: 76 bp | Trich_R |  | CTGATTCTCCGTTAACCGTTGTC |
|  | Trich_P | FAM-BHQ1 | CGATGGTACGCTACGTGCTTACCATGG |
| **Ancylostoma duodenale qPCR** | Ancyl_F |  | GAATGACAGCAAACTCGTTGTTG |
| Target: ITS, Size: 71 bp | Ancyl_R |  | ATACTAGCCACTGCCGAAACGT |
|  | Ancyl_P MGB | HEX-MGB-EQ | ATCGTTTACCGACTTTAG |
| **Necator americanus qPCR** | Neca_F |  | TGTTTGTCGAACGGTACTTGYTCT |
| Target: ITS, Size: 100 bp | Neca_R1 |  | GTGAATAACAGCGTGCACATGTTG |
|  | Neca_R2 |  | CTGACAATGACAGTTTGCATATGTTG |
|  | Neca_P MGB | HEX-MGB-EQ | ATTCCCGTTTAAGTGAAGA |
| **Strongyloides stercoralis qPCR** (generic) | S28S_F |  | GCGAACAAGTACTGTGAAGGAAAATTG |
| Target: 28S, Size:  92 bp | S28S_R |  | TGGCTCTGTATGCTTCCATCGT |
|  | S28S_p | HEX-BHQ1 | CCGGAGAGAGAGTTAAAGAGGACGT |
