# Supplementary material for: Performance of the Kato-Katz method and real time polymerase chain reaction for the diagnosis of soil-transmitted helminthiasis in the framework of a randomised controlled trial: treatment efficacy and day-to-day variation
Source: Parasit Vectors. 2020 Oct 15;13:517. doi: 10.1186/s13071-020-04401-x (PMC7558729; doi:10.1186/s13071-020-04401-x)
Supplement: Supplementary file 2 — Additional file 2: Table S2. GeneExpression MasterMix. [file 13071_2020_4401_MOESM2_ESM.docx]

**Additional file 2: Table S2. GeneExpression MasterMix.**

|  | Concentration | Per reaction of 25 µl (µl) |
| --- | --- | --- |
|  |  |  |
|  |  |  |
|  |  |  |
|  |  |  |
|  |  |  |
| ddH2O |  | 3.901 |
| GeneEx Master Mix | 2x | 12.5 |
| Asca_F | 50µM | 0.1 |
| Asca_R | 50µM | 0.1 |
| Trich_F | 50µM | 0.2 |
| Trich_R | 50µM | 0.2 |
| Ancyl_F | 50µM | 0.2 |
| Ancyl_R | 50µM | 0.2 |
| Nec_F | 50µM | 0.133 |
| Nec_R1 | 50µM | 0.133 |
| Nec_R2 | 50µM | 0.133 |
| Ss_F | 50µM | 0.2 |
| Ss_R | 50µM | 0.2 |
| Probe Asc_P (FAM) | 10µM | 0.2 |
| ProbeTri_P (Cy5) | 10µM | 0.4 |
| Probe Ancyl_P (HEX) | 10µM | 0.4 |
| Probe Nec_P (HEX) | 10µM | 0.4 |
| Probe SsI_P (HEX) | 10µM | 0.4 |
| Subtotal |  | 20 |
| DNA |  | 5 µl |
